# Survival Outcomes in Pancreatic Neuroendocrine Tumors: A Systematic Review and Meta-Analysis of Progression-Related Endpoints

**DOI:** 10.3390/cancers18111705

**Published:** 2026-05-23

**Authors:** Lavinia Simona Neculai-Candea, Andreea-Daniela Caloian, Sorin Deacu, Miruna Cristian, Laura Mazilu, Andreea-Corina Ilie-Petrov, Radu Adrian Nitu, Carmen Aida Ciufu, Nicolae Ciufu

**Affiliations:** 1 Department of Forensic Medicine, “Sf. Apostol Andrei” Emergency County Hospital, 900439 Constanta, Romania; lavinia.candea@365.univ-ovidius.ro (L.S.N.-C.); miruna.cristian@365.univ-ovidius.ro (M.C.); 2 Legal Medicine Pre-Clinics Department, University Ovidius Constanta, 905900 Constanta, Romania; 3 Department of Oncology and Hematology, Faculty of Medicine, Ovidius Clinical Hospital, University Ovidius Constanta, 905900 Constanta, Romania; 4 Department of Histology, University Ovidius Constanta, 905900 Constanta, Romania; 5 Center for Research and Development of the Morphological and Genetic Studies of Malignant Pathology-CEDMOG, “Ovidius” University of Constanta, 900470 Constanta, Romania; 6 Department of Pathology, St. Vincent’s University Hospital, Elm Park, D04 T6F4 Dublin, Ireland; 7 Department of Cardiovascular Surgery, County Clinical Emergency Hospital of Constanta, 900439 Constanta, Romania; radu_nitzu@yahoo.com; 8 Department of Radiology, Faculty of Medicine, Ovidius Clinical Hospital, University Ovidius Constanta, 905900 Constanta, Romania; 9 Department of Surgery, Faculty of Medicine, Ovidius Clinical Hospital, University Ovidius Constanta, 905900 Constanta, Romania; nicolae.ciufu@365.univ-ovidius.ro

**Keywords:** pancreatic neuroendocrine tumors, neuroendocrine neoplasms, progression-free survival, overall survival, recurrence-free survival, systematic review, meta-analysis, targeted therapy, peptide receptor radionuclide therapy, pancreatic surgery

## Abstract

**Background:** Pancreatic neuroendocrine tumors (pNETs) represent a heterogeneous group of neoplasms characterized by variable biological behavior and clinical outcomes. Multiple therapeutic strategies have been investigated, including surgery, targeted therapies, peptide receptor radionuclide therapy, and systemic treatments. The present study aimed to summarize survival-related outcomes reported across studies investigating the management of pNETs. **Methods:** A systematic review of the literature was conducted including studies reporting clinical outcomes in patients with pancreatic neuroendocrine tumors. A total of 27 studies were included in the qualitative analysis. Survival-related outcomes, such as progression-free survival (PFS), recurrence-free survival (RFS), and recurrence rates, were extracted. Studies reporting quantitative survival values were included in the meta-analytical component. A random-effects model was applied, and a forest plot was generated to summarize the reported outcomes. **Results:** Reported survival outcomes varied substantially across studies. Median PFS values ranged from approximately 5.6 to 86.5 months, while several surgical series reported 5-year overall survival rates exceeding 90%. Recurrence rates following surgical resection ranged from approximately 12% to 26% in some cohorts. The pooled estimate derived from the meta-analytical model was 32.22 (95% CI: 15.65–48.80). **Conclusions:** The analysis summarizes survival-related outcomes reported in studies investigating pancreatic neuroendocrine tumors and provides a quantitative overview of the reported progression-related endpoints across the analyzed literature.

## Simple Summary

Pancreatic neuroendocrine tumors are rare neoplasms with heterogeneous clinical behavior and variable treatment outcomes. Several therapeutic strategies have been reported, including surgery, systemic therapy, targeted agents, and peptide receptor radionuclide therapy. This study reviews published studies reporting survival outcomes in patients with pancreatic neuroendocrine tumors. Progression-related outcomes were extracted and analyzed using a meta-analytical approach to provide a quantitative overview of survival outcomes reported in the current literature.

## 1. Introduction

Neuroendocrine tumors represent a heterogeneous group of neoplasms arising from neuroendocrine cells located throughout the body, particularly within the gastrointestinal tract, pancreas, and lungs [1]. These tumors are characterized by their ability to synthesize and secrete biologically active peptides and hormones, resulting in diverse clinical manifestations depending on the tumor’s functional status and the type of secreted hormone [2]. pNETs demonstrate substantial variability in biological behavior, ranging from slow-growing, indolent tumors to aggressive neoplasms with significant metastatic potential [3]. Insulinomas represent the most common functional pancreatic neuroendocrine tumors and arise from insulin-secreting β-cells located within the islets of Langerhans [4].

These tumors are typically small, well-differentiated neoplasms characterized by inappropriate insulin secretion, which leads to recurrent episodes of hypoglycemia [5]. Although most insulinomas are benign and solitary, a minority may exhibit malignant behavior with metastatic potential [6].

**Figure 1:**
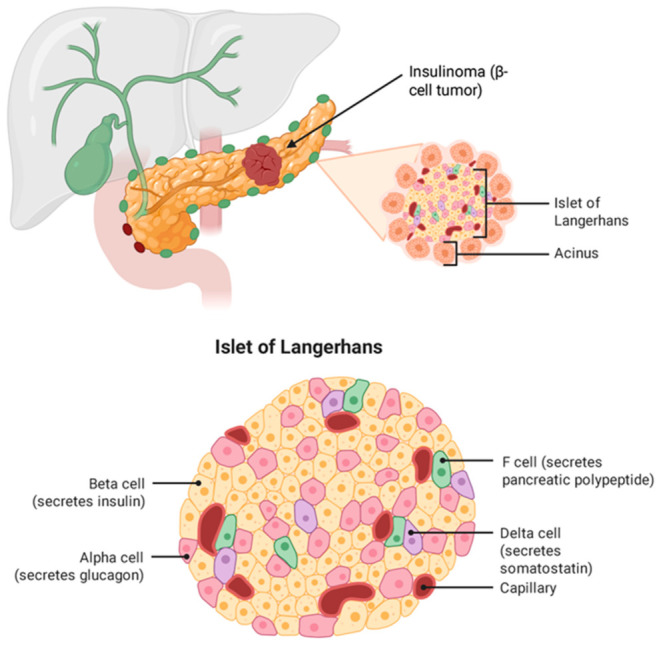
Anatomical localization of insulinoma and cellular organization of the pancreatic islets. The **upper** panel illustrates the anatomical position of the tumor within the pancreas and the relationship between pancreatic acini and the islets of Langerhans. The **lower** panel depicts the cellular composition of the pancreatic islet, including β-cells (insulin secretion), α-cells (glucagon secretion), δ-cells (somatostatin secretion), and pancreatic polypeptide (F) cells, surrounded by a dense capillary network. Created in BioRender. Twakor, A. (2026) https://BioRender.com/0bgfitv, accessed on 11 April 2026. [7].

Figure 1 created with Biorender [7] is a schematic representation of the pancreas showing the localization of an insulinoma arising from pancreatic β-cells [8].

A descriptive analysis of the global epidemiological indicators of pancreatic tumors is shown in Table 1.

The mean age-standardized rate (ASR World) was 4.3 cases per 100,000 population, with a median value of 3.8. The crude incidence rate demonstrated a higher mean value of 7.6 cases per 100,000, with a range reaching 37.6. The cumulative risk of developing insulinoma was 0.50.

We also performed a Spearman’s correlation analysis (Table 2) between ASR (World), crude rate, cumulative risk, and number of cases (*N* = 186).

The correlation coefficients for ASR (World) with crude rate and cumulative risk were ρ = 0.961 (*p* < 0.001) and 0.994 (*p* < 0.001), respectively, which are very strong positive correlations. The correlation coefficient between crude rate and cumulative risk was ρ = 0.954 (*p* < 0.001), which is a very strong positive correlation as well. Moderate correlations were noted for number of cases and ASR (World) (ρ = 0.520, *p* < 0.001), number of cases and crude rate (ρ = 0.540, *p* < 0.001), and number of cases and cumulative risk (ρ = 0.522, *p* < 0.001). All correlations were statistically significant at *p* < 0.01.

According to the 2010 World Health Organization’s (WHO, Geneva, Switzerland) Classification of Tumors of the Digestive System, pNETs are categorized into three histological grades based on mitotic activity and Ki-67 proliferative index. Grade 1 (G1) tumors are characterized by fewer than 2 mitoses per 10 high-power fields (HPF) or a Ki-67 index below 3%, whereas Grade 2 (G2) tumors demonstrate 2–20 mitoses per 10 HPF or a Ki-67 index ranging from 3% to 20% [11]. Grade 3 (G3) neoplasms exhibit more than 20 mitoses per 10 HPF or a Ki-67 index exceeding 20%. G1 and G2 tumors are generally considered well-differentiated neoplasms, while G3 tumors are classified as poorly differentiated high-grade neuroendocrine carcinomas, including both small-cell and large-cell neuroendocrine carcinoma subtypes [12]. This grading system was developed in response to accumulating evidence demonstrating a strong association between tumor proliferative activity and clinical outcomes [13].

The clinical presentation of NETs varies widely depending on tumor location, functional status, and tumor burden. Functional tumors may produce hormones responsible for characteristic endocrine syndromes, whereas non-functional tumors frequently remain clinically silent until they reach an advanced stage or metastasize [14]. Among patients with gastroenteropancreatic NETs, the liver represents the most common site of metastatic disease [15]. Diagnosis is typically established through a combination of biochemical markers, imaging techniques, and histopathological confirmation. Circulating biomarkers, such as chromogranin A, are commonly used in clinical practice for disease detection and monitoring [16]. Imaging modalities, including computed tomography, magnetic resonance imaging, and somatostatin receptor–based positron emission tomography, are widely employed for tumor localization, staging, and treatment planning [17].

Management of NETs is determined by tumor grade, stage, and functional status. Surgical resection remains the primary treatment modality for localized disease and is associated with improved survival outcomes in selected patients [18]. However, a substantial proportion of patients are diagnosed with unresectable or metastatic disease, necessitating systemic therapeutic approaches [19]. Available treatment strategies include somatostatin analogs, targeted therapies, chemotherapy, and peptide receptor radionuclide therapy (PRRT) [20]. Somatostatin analogs, such as lanreotide, are widely used to control hormone-related symptoms and to stabilize tumor growth in patients with well-differentiated NETs [21]. In addition, targeted therapeutic agents and combination treatment regimens have demonstrated activity in advanced pancreatic NETs and represent important options in progressive disease [22].

Despite advances in diagnostic and therapeutic strategies, the management of NETs remains complex because of their biological heterogeneity and variable clinical course. The relatively low incidence of these tumors and the diversity of their clinical presentation limit the availability of large randomized clinical trials and contribute to variability in reported outcomes across treatment modalities [23].

## 2. Materials and Methods

### 2.1. Study Design

This meta-analysis was conducted in accordance with the Preferred Reporting Items for Systematic Reviews and Meta-Analyses (PRISMA) guidelines [24]. The objective of the study was to evaluate the efficacy and safety of various treatment modalities for pancreatic neuroendocrine tumors, including surgery, chemotherapy, targeted therapies, and PRRT. The study protocol was prospectively registered in the International Prospective Register of Systematic Reviews (PROSPERO) under registration number CRD420261334158. [25].

### 2.2. Eligibility Criteria

Our examination included peer-reviewed randomized controlled trials (RCTs), cohort studies, and clinical trials.

Included studies were solely published in English and the search time frame was set between January 2021 to February 2026. We also manually searched the references to identify other potentially helpful articles missed by our search strategy.

Eligible studies were selected according to predefined inclusion and exclusion criteria established before the initiation of data extraction and analysis. Studies were considered eligible if they reported progression-related outcomes in patients with pancreatic neuroendocrine tumors and provided sufficient clinical or survival-related data for qualitative or quantitative assessment. Both prospective and retrospective observational studies, as well as randomized clinical trials, were considered eligible due to the limited availability of homogeneous large-scale studies addressing progression-related outcomes in pNETs.

However, the inclusion of studies with different methodological designs and heterogeneous patient populations may represent an important source of clinical and methodological heterogeneity. Variability in tumor grading, disease stage, metastatic burden, treatment modalities, and progression-related endpoint definitions may have influenced pooled effect estimates and limited direct comparability among studies. Although subgroup analyses and meta-regression were considered, the relatively limited number of studies with sufficiently homogeneous reporting reduced the statistical robustness of such analyses. Consequently, pooled analyses were interpreted cautiously, and the findings should primarily be considered exploratory and hypothesis-generating [26].

The literature search strategy was expanded to improve comprehensiveness and minimize the risk of incomplete study identification. In addition to PubMed [27], supplementary searches were conducted using the Cochrane Library [28] and Web of Science databases. ScienceDirect [29] was additionally used as a supplementary source for article retrieval and cross-referencing when applicable. The additional database screening did not identify studies that substantially altered the final study selection or pooled analyses. The overall search strategy was conducted according to predefined eligibility criteria and PRISMA recommendations to ensure methodological transparency and reproducibility.

### 2.3. Search Strategy

Boolean operators [30] were used to combine keywords, and filters were applied to limit results to human studies and publications in English:

pNETs: “pancreatic neuroendocrine tumors” OR “pNETs” OR “carcinoid” OR “gastroenteropancreatic neuroendocrine tumors” OR “neuroendocrine tumors.”

Treatment: “treatment” OR “therapy” OR “surgery” OR “chemotherapy” OR “targeted therapy” OR “radiotherapy.” The full list of citations is included in Supplementary Materials.

### 2.4. Study Selection

All studies were screened and selected according to predefined inclusion and exclusion criteria established before the initiation of data extraction and analysis. Eligible studies included original investigations reporting progression-related outcomes in patients with pancreatic neuroendocrine tumors, whereas studies lacking sufficient outcome data, non-English publications, conference abstracts without full text, duplicate reports, review articles, editorials, and case reports were excluded. The literature screening and eligibility-assessment process was conducted independently in order to minimize subjective selection and reduce the risk of selection bias. Any discrepancies identified during study selection were resolved through discussion and consensus.

The extracted information included study characteristics, such as authors, year of publication, study design, and sample size. Participant characteristics included age, sex, tumor type, and tumor grade. Intervention-related information included treatment type, duration, and follow-up period. Outcome data included primary and secondary outcomes, together with reported effect measures, such as hazard ratios or relative risks, and statistical significance when available.

The risk of bias for randomized controlled trials was evaluated using the Cochrane Risk of Bias Tool version 2 [31].

### 2.5. Software and Statistical Analysis

Statistical analyses were performed using SPSS software (version 29.0; IBM Corp., Armonk, NY, USA) [9]. Meta-analyses and forest plots were generated using the same software. All statistical tests were two-sided, and statistical significance was defined as a *p*-value < 0.05 [32]. The review was conducted and reported according to the PRISMA guidelines. The main findings are presented in the text and summarized in tables, while forest plots are used to display the results of the meta-analyses. Statistical heterogeneity among pooled studies was assessed using Cochran’s Q test and the I^2^ statistic. An I^2^ value greater than 50% was considered indicative of substantial heterogeneity. Due to the expected clinical and methodological variability among included studies, pooled analyses were performed using random-effects models.

### 2.6. PRISMA Framework

**Figure 2:**
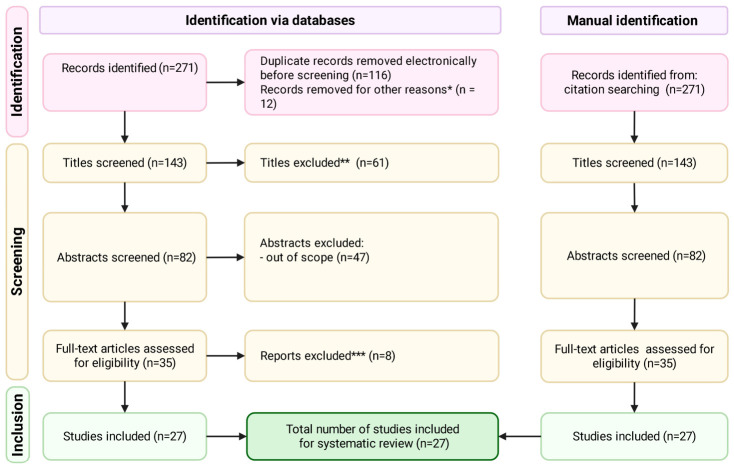
PRISMA framework. Created in BioRender. Twakor, A. (2026) https://BioRender.com/7m3u6po. * Studies were not relevant to the present review/** Studies did not help provide an answer to the current research/*** Abstracts were not relevant for the research.

A total of 271 records were identified through database searching. In addition, 271 records were identified through manual citation searching. Prior to screening, 116 duplicate records were removed electronically, and 12 records were excluded for other reasons, leaving 143 records for title screening. During the title screening stage, 61 records were excluded because they did not meet the predefined inclusion criteria. The remaining 82 records proceeded to abstract screening, where 47 studies were excluded because they were outside the scope of the present research. Subsequently, 35 full-text articles were assessed for eligibility. After full-text evaluation, 8 reports were excluded due to failure to meet the inclusion criteria or insufficient relevant data. As a result, 27 studies were included in the final systematic review, as illustrated in the PRISMA flow diagram (Figure 2).

### 2.7. Risk of Bias Assessment

The methodological quality of the included observational studies was assessed using the Newcastle–Ottawa Scale (NOS). This validated tool evaluates studies across three domains: selection of study groups, comparability of cohorts, and adequacy of outcome assessment. Studies were assigned a maximum of nine points, with scores of 7–9 considered high quality, 5–6 considered moderate quality, and below 5 considered low quality. The quality assessment was performed independently for all eligible studies, and a summary of the NOS scores is included in the Supplementary Material.

## 3. Results

### Pancreatic N.E.T.s

A total of 27 studies investigating the management and outcomes of pancreatic neuroendocrine tumors are included in Table 3, comprising randomized trials, prospective studies, and retrospective cohort analyses from multiple international centers [33,34,35,36,37,38,39,40,41,42,43,44,45,46,47,48,49,50,51,52,53,54,55,56,57,58,59].

Sample sizes varied widely across the included studies, ranging from small exploratory trials with only a few patients to large multicenter cohorts including more than 800 participants [43,46,52,59]. Most studies included adult patients with median ages between approximately 55 and 67 years and relatively balanced sex distributions. Tumors were predominantly located in the pancreas, although several investigations also included gastroenteropancreatic or extra-pancreatic NETs [35,36,45,50,54,57]. The evaluated therapeutic strategies were heterogeneous and comprised surgical resection, systemic targeted therapies, chemotherapy, PRRT, immunotherapy, and minimally invasive approaches. Addeo et al. compared minimally invasive with open distal pancreatectomy [33], Capdevila et al. evaluated sequential systemic therapies [34], and cabozantinib efficacy was assessed in randomized trials by Chan et al. and Strosberg et al. [35,57]. Other studies focused on surgical prognostic factors and treatment algorithms [46,55,59].

The included studies demonstrated considerable heterogeneity in treatment strategies and outcomes. Surgical resection remains the cornerstone of treatment for localized disease, with several cohorts reporting 5-year overall survival rates exceeding 90% [36,46,56]. Minimally invasive techniques showed comparable oncologic outcomes with reduced morbidity [33,38]. Prognostic factors such as lymph node involvement, tumor grade, and tumor size were consistently associated with recurrence and survival [55,59]. In advanced disease, targeted therapies such as everolimus and cabozantinib showed improved disease control [34,35,53], while newer approaches, including PRRT and immunotherapy, are being explored in smaller or early-phase studies [39,45,52].

Several investigations were retrospective multicenter cohort studies evaluating surgical outcomes and prognostic factors following pancreatic resection [33,36,37,38,40,46,47,49,55,56,58,59], whereas other studies consisted of prospective or randomized clinical trials investigating systemic therapies, targeted agents, immunotherapy, or peptide receptor radionuclide therapy (PRRT) [34,35,39,43,44,45,51,52,53,54,57]. Surgical interventions included minimally invasive distal pancreatectomy, pancreaticoduodenectomy, spleen-preserving resections, hepatic metastasectomy, and curative-intent resections with lymphadenectomy [33,36,38,40,46,47,55,56,58,59]. Systemic treatment strategies involved everolimus, streptozotocin plus 5-fluorouracil, cabozantinib, lanreotide, FOLFOX chemotherapy, entinostat, dual immune checkpoint blockade, and MAP-guided targeted therapies [34,35,42,43,44,50,52,53,54,57]. Additional studies evaluated novel or minimally invasive approaches, including neoadjuvant PRRT, dosimetry-guided radionuclide therapy, belzutifan therapy, and EUS-guided ethanol injection for small pNETs [39,45,48,51].

**Figure 3:**
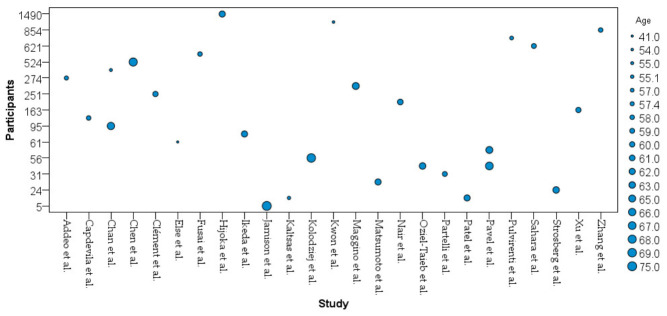
Distribution of study sample size and age across the included studies [33,34,35,36,37,38,39,40,41,42,43,44,45,46,47,48,49,50,51,52,53,54,55,56,57,58,59]. Created with SPSS version 30.0 [10]. Raw data are contained in Supplementary Materials.

Figure 3 presents the number of participants and male patients reported in each included study.

Sample sizes ranged from 5 participants in Jamison et al. [43] to 1490 participants in Hijoka et al. [41]. Other large cohorts included Kwon et al. [46] (918 participants), Zhang et al. [59] (854), Pulvirenti et al. [55] (632), Sahara et al. [56] (621), Fusai et al. [40] (541), and Chen et al. [37] (524). Smaller studies included Kaltsas et al. [44] (19), Patel et al. [52] (19), Matsumoto et al. [48] (25), Partelli et al. [51] (31), and Oziel-Taieb et al. [50] (48). The number of male participants ranged from 4 in Jamison et al. [43] to 978 in Hijoka et al. [41], with other studies reporting between 10 and 486 male patients.

The Pearson’s correlation analysis (Table 4) showed a statistically significant positive correlation between the number of participants and PFS (r = 0.592, *p* = 0.026; N = 14).

The correlation between participants and OS was not statistically significant (r = −0.054, *p* = 0.909; *N* = 7). Similarly, the correlation between PFS and OS was not statistically significant (r = 0.245, *p* = 0.640; *N* = 6). Correlations were calculated using data from 27 studies reporting participant numbers [33,34,35,36,37,38,39,40,41,42,43,44,45,46,47,48,49,50,51,52,53,54,55,56,57,58,59], 14 studies reporting PFS [33,34,35,36,38,40,41,42,44,48,50,53,54,57], and 7 studies reporting OS outcomes [33,34,35,42,48,50,58].

The Spearman’s correlation analysis in Table 5 demonstrates a statistically significant positive correlation between the number of participants and PFS (ρ = 0.744, *p* = 0.002; *N* = 14).

The correlation between participants and OS was not statistically significant (ρ = 0.214, *p* = 0.645; *N* = 7). The correlation between PFS and OS was also not statistically significant (ρ = 0.600, *p* = 0.208; *N* = 6). Correlations were calculated using data from 27 studies reporting participant numbers [33,34,35,36,37,38,39,40,41,42,43,44,45,46,47,48,49,50,51,52,53,54,55,56,57,58,59], 14 studies reporting PFS [33,34,35,36,38,40,41,42,44,48,50,53,54,57], and 7 studies reporting OS outcomes [33,34,35,42,48,50,58].

**Figure 4:**
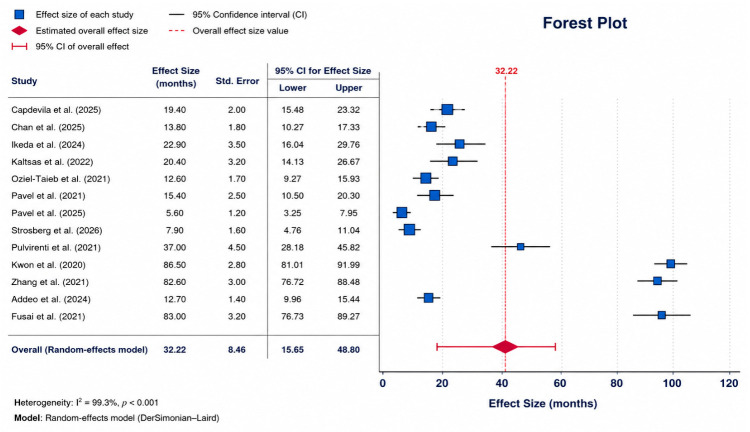
Forest plot of pooled progression-related outcomes across included studies [33,34,35,40,42,44,46,50,53,54,55,57,59].

In Figure 4, each study is represented by a point estimate (effect size) and its corresponding 95% confidence interval derived from the reported standard error.

The individual effect size values ranged from 5.6 to 86.5, with corresponding 95% confidence intervals displayed for each study. The smallest effect size was reported in Pavel et al. (2025) [54], with a value of 5.6 (95% CI 3.3–7.9), while the largest effect size was reported in Kwon et al. [46], with a value of 86.5 (95% CI 81.0–92.0). Several studies reported values between approximately 12 and 23, including Capdevila et al. [34], Chan et al. [35], Ikeda et al. [42], Kaltsas et al. [44], Oziel-Taieb et al. [50], and Pavel et al. [53]. Higher values were reported in Pulvirenti et al. [55], Zhang et al. [59], and Fusai et al. [40]. Considerable heterogeneity was observed among included studies (I^2^ = 99.3%, *p* < 0.001), supporting the use of a random-effects model for pooled analyses.

Clinically, the pooled estimate of 32.22 (95% CI: 15.65–48.80) represents the average progression-related outcome across the included studies, calculated by combining the reported values using a random-effects meta-analytic model. In practical terms, this value reflects the overall central tendency of progression-related survival outcomes (PFS or RFS) reported in the analyzed cohorts of patients. The included studies evaluated a wide range of therapeutic approaches, including surgical resection, targeted therapies, chemotherapy, peptide receptor radionuclide therapy, immunotherapy, and minimally invasive interventions, which may have contributed to the broad dispersion of effect sizes observed in the forest plot. In addition, variability in follow-up duration, metastatic burden, and progression assessment criteria further limited direct comparability between studies. Therefore, a random-effects meta-analytical model was applied in order to account for both clinical and methodological heterogeneity among the included investigations.

**Figure 5:**
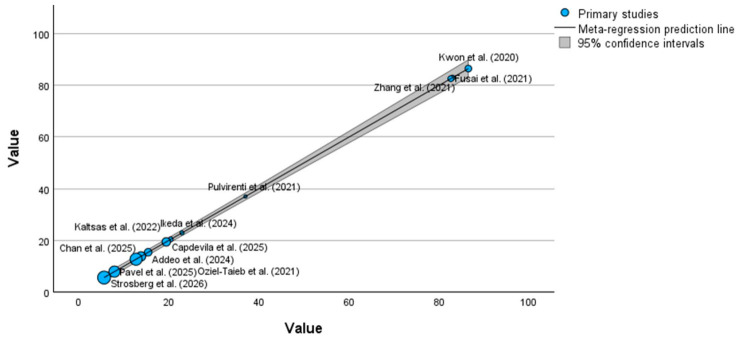
Meta-regression plot of progression-related outcomes across studies of pNETs [33,34,35,40,42,44,46,50,53,54,55,57,59].

The meta-regression plot in Figure 5 illustrates the distribution of progression-related outcome values reported across the included studies.

The individual study values ranged from 5.6 to 86.5. The lowest values were observed in Strosberg et al. [57], Pavel et al. [54], and Chan et al. [35], where the reported values were below 15. Intermediate values of between approximately 15 and 25 were reported in Capdevila et al. [34], Oziel-Taieb et al. [50], Ikeda et al. [42], Kaltsas et al. [44], and Addeo et al. [33]. Higher values were observed in Pulvirenti et al. [55], while the largest values were reported in Kwon et al. 46], Zhang et al. [59], and Fusai et al. [40], where the values exceeded 80.

The analysis of the included studies demonstrated a wide distribution of progression-related outcomes reported in patients with pNETs. PFS values ranged from 5.6 to 86.5 months, with lower values observed in Pavel et al. [54], Strosberg et al. [57], and Chan et al. [35], while higher values were reported in Kwon et al. [46], Zhang et al. [59], and Fusai et al. [40]. The random-effects meta-analysis yielded a pooled estimate of 32.22 (95% CI: 15.65–48.80) for progression-related outcomes across the analyzed studies. Additionally, correlation analysis identified a significant positive association between the number of participants and reported PFS values (ρ = 0.744, *p* = 0.002), whereas no statistically significant correlations were observed between participant number and OS or between PFS and OS.

**Figure 6:**
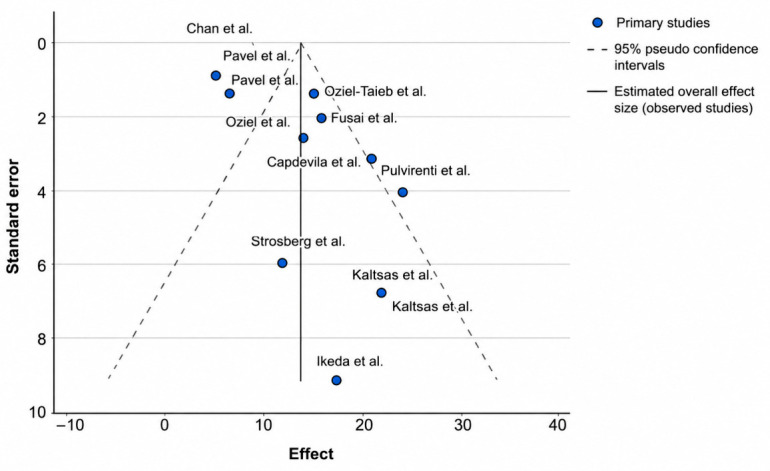
Funnel plot assessing publication bias among studies included in the meta-analysis [33,34,35,40,42,44,46,50,53,54,55,57,59].

Figure 6 shows a mild asymmetry in the distribution of included studies around the estimated overall effect size.

Smaller studies with larger standard errors appeared more widely dispersed, whereas studies with lower standard errors clustered closer to the pooled estimate. This pattern may suggest the presence of small-study effects or potential publication bias; however, interpretation is limited by the relatively small number of included studies and the substantial methodological and clinical heterogeneity among studies, including differences in study design, treatment modalities, and reported progression-related outcomes.

## 4. Discussion

The results of the present analysis demonstrate a broad range of survival outcomes across studies evaluating therapies for pNETs. Similar variability in clinical outcomes has been reported in multiple previously published investigations. For instance, Kunz et al. reported in a randomized phase II trial that the combination of capecitabine and temozolomide significantly improved PFS compared with temozolomide monotherapy in patients with advanced pNETs, with a median PFS of 22.7 months versus 14.4 months, respectively (HR 0.58) [60].

The substantial heterogeneity observed across the included studies is likely multifactorial and reflects the intrinsic complexity of pancreatic neuroendocrine tumors as well as the variability in study methodology. Important potential sources of heterogeneity include differences in study design, as the analysis incorporated randomized controlled trials, prospective observational studies, and retrospective cohort analyses with variable levels of methodological rigor. Additional heterogeneity may arise from differences in patient populations, including variations in tumor grade (G1–G3), disease stage, metastatic burden, functional versus non-functional tumors, and prior treatment exposure. Considerable variability was also identified in therapeutic strategies, with studies evaluating surgical resection, chemotherapy, targeted therapies, peptide receptor radionuclide therapy, immunotherapy, and minimally invasive approaches. Furthermore, progression-related endpoints were not uniformly defined across studies, as some investigations reported progression-free survival while others reported recurrence-free survival or recurrence-related outcomes. Variability in follow-up duration, sample size, and treatment sequencing may have additionally contributed to differences in the magnitude and direction of reported estimates.

Similarly, Grande et al. reported meaningful clinical activity with targeted therapeutic approaches in patients with advanced pNETs [61]. Comparable observations were described by Kulke et al., who reported significant improvements in disease control with systemic therapies in advanced pNETs [62].

In addition, Kunz et al. demonstrated that biomarkers such as MGMT deficiency may be associated with improved response to temozolomide-based therapy [60].

Other studies included in the present comparison further emphasize the heterogeneity of therapeutic responses. Imam et al. reported variable treatment responses across different therapeutic strategies in patients with neuroendocrine tumors [63]. Similar patterns were described by Bolm et al. and Paik et al., who highlighted the importance of tumor biology and disease stage in determining clinical outcomes and survival trajectories in patients with neuroendocrine neoplasms [64,65].

Patients with advanced or metastatic disease frequently require multimodal therapeutic strategies integrating surgery, systemic therapies, targeted agents, liver-directed therapies, and peptide receptor radionuclide therapy. In this context, Citterio et al. [66] reported that multimodal treatment approaches were associated with prolonged survival and improved disease control in patients with advanced neuroendocrine tumors, emphasizing the importance of individualized therapeutic sequencing and multidisciplinary management in optimizing long-term outcomes. Our findings are consistent with these observations, as the studies included in the present meta-analysis demonstrated substantial variability in progression-free survival according to the therapeutic strategy employed. Patients undergoing combined or targeted therapeutic approaches generally demonstrated longer progression-related outcomes compared with those treated with single-modality systemic therapy alone.

Also, recent evidence from the meta-analysis by Armellini et al. [67] demonstrated high technical and clinical success rates for EUS-guided ablative therapies in small pNETs, with favorable safety profiles and low rates of severe adverse events. This evolving therapeutic trend, as several studies included in the present analysis highlighted, show the growing role of minimally invasive and multimodal approaches in improving progression-related outcomes while limiting treatment-related morbidity.

## 5. Limitations

The included studies evaluated a broad spectrum of therapeutic methodologies, including surgical resection, targeted therapies, chemotherapy, peptide receptor radionuclide therapy, immunotherapy, and minimally invasive interventions. This therapeutic variability reflects the complex and heterogeneous nature of pancreatic neuroendocrine tumors and represents an important source of clinical heterogeneity within the present analysis. In addition, substantial differences were identified in study design, patient populations, tumor grading, metastatic burden, treatment sequencing, and definitions of progression-related endpoints. These factors may limit the direct comparability of survival outcomes across studies and reduce the ability to establish a single consensus therapeutic conclusion. Therefore, the primary objective of the present study was not to determine the superiority of one treatment modality over another but rather to provide a comprehensive overview of progression-related outcomes reported in the contemporary literature on pancreatic neuroendocrine tumors. Consequently, the findings of this meta-analysis should be interpreted primarily as exploratory and descriptive, while highlighting the need for future large-scale prospective studies with more homogeneous patient populations and standardized outcome reporting.

Not all studies reported identical survival endpoints, as some reported PFS while others reported recurrence-free survival or other related outcomes, requiring the combination of these endpoints within the same analysis. In addition, the extracted data were derived from published studies rather than individual patient-level datasets.

Also, the substantial variability in tumor grading, staging distribution, and metastatic burden across the included studies may have influenced pooled effect estimates and contributed to methodological heterogeneity. Differences in the proportion of low-grade versus high-grade tumors, localized versus metastatic disease, and functional versus nonfunctional pNETs may significantly affect progression-related outcomes and limit the comparability of survival estimates between studies.

Finally, variations in follow-up duration and reporting formats across the included studies may influence the comparability of the reported survival values and response outcomes.

## 6. Conclusions

The present study analyzed survival outcomes reported across multiple studies investigating treatments for neuroendocrine tumors.

The forest plot analysis summarized the effect sizes reported across these studies using a random-effects model. Individual effect size values ranged from 5.6 to 86.5 across the included studies. The pooled estimate calculated using the random-effects model was 32.22, with a 95% confidence interval of 15.65–48.80. The graphical representation of the results showed the effect size for each study together with the corresponding confidence intervals and the pooled estimate.

The analysis incorporated survival-related outcomes reported across the included studies, including PFS, recurrence-free survival, and related survival endpoints. These outcomes were extracted from the reported results of each study and incorporated into the meta-analytical framework to allow a combined summary estimate. The results presented in the forest plot provide a quantitative summary of the survival outcomes reported in the included studies and illustrate the distribution of reported effect sizes across the analyzed literature.

## Figures and Tables

**Table 1:** Descriptive statistics of global epidemiological indicators for pancreatic tumor incidence. Raw data were obtained from the International Agency for Research on Cancer [9] and are contained in Supplementary Material. Table created with IBM-SPSS version 30.0 [10].

	Mean	Median	Range
ASR (World)	4.3	3.8	
Crude rate	7.6		37.6
Cumulative risk	0.50		

**Table 2:** Spearman correlation analysis of epidemiological indicators for insulinoma incidence. Table created with IBM-SPSS version 30.0 [10]. Raw data were obtained from the International Agency for Research on Cancer [9] and are contained in Supplementary Material S1.

			ASR (World)	Crude Rate	Cumulative Risk	Number
Spearman’s rho	ASR (World)	Correlation Coefficient	1.000	0.961 **	0.994 **	0.520 **
		Sig. (2-tailed)	.	<0.001	<0.001	<0.001
		N	186	186	186	186
	Crude rate	Correlation Coefficient	0.961 **	1.000	0.954 **	0.540 **
		Sig. (2-tailed)	<0.001	.	<0.001	<0.001
		N	186	186	186	186
	Cumulative risk	Correlation Coefficient	0.994 **	0.954 **	1.000	0.522 **
		Sig. (2-tailed)	<0.001	<0.001	.	<0.001
		N	186	186	186	186
	Number	Correlation Coefficient	0.520 **	0.540 **	0.522 **	1.000
		Sig. (2-tailed)	<0.001	<0.001	<0.001	.
		N	186	186	186	186

**. Correlation is significant at the 0.01 level (2-tailed).

**Table 3:** Characteristics of selected studies.

Study	Country	Participants (*n* = )	Age Median	Sex Distribution	Tumor Site	Tumor Classification	PFS	OS	Treatment Type/Duration	Key Findings	Limitations
Addeo et al., 2024 [33]	France	274	57.4 years (18–85.7)	Male/Female: 129/145	Pancreas (distal pancreas)	pNETs (G1–G3)	75.7 months; recurrence rate 12.7%	5-year OS: 96.2% overall (79% MIS vs. 75% open after PSM)	Minimally invasive distal pancreatectomy vs. open distal pancreatectomy, median operative time: 180 min.	Minimally invasive surgery reduced major morbidity and delayed gastric emptying with comparable oncologic outcomes to open surgery	Retrospective design; inter-center variability; limited matched sample size
Capdevila et al., 2025 [34]	Spain, Germany, France, Denmark, the Netherlands, Sweden, Italy, UK	141	58 years (33–83)	Male/Female: 85/56	Pancreas	pNETs mainly G1–G2	19.4 months (everolimus first) vs. 22.7 months (STZ/5-FU first)	61.7 months (everolimus first) vs. 50.6 months (STZ/5-FU first)	Sequential systemic therapy: everolimus vs. streptozotocin + 5-fluorouracil (STZ/5-FU), median follow-up: 36.6 months	STZ/5-FU produced higher response rates, while PFS and OS were not significantly different between treatment sequences	Moderate sample size; limited power to detect OS differences
Chan et al., 2025 [35]	USA	203 extra-pancreatic tumors, 95 pNETs	66 years (28–86) Cabozantinib, 66 (30–82) placebo	Male/Female: 98/105	Extra and pancreatic tumors	pNETs mainly G2–G3	13.8 months vs. 4.4 months (placebo)	40 months vs. 31.1 months (placebo)	Cabozantinib vs. placebo, duration 3.8 months in pNETs group	Cabozantinib improves PFS in patients with previously treated advanced extra-pancreatic or pNETs.	Early trial termination could lead to overestimation of treatment effect
Chan et al., 2021 [36]	USA	294 GEP-NETs out of with 141 with pNETs (2004–2008)	55 years (20–90)	Male/Female: 149/145	Gastroenteropancreatic (GEP) tract	Well- or moderately differentiated GEP-NETs	pNETs group: 52.1 months for patients with metastasis, 97.4 months in those with regional disease	5-year OS: 92% (small bowel), 94% (pancreas), 96% (colon/rectum)	Surgical resection with curative intent (R0 resection of primary tumor ± metastasectomy), median follow-up: 62.1 months	R0 surgical resection was associated with high long-term survival, but recurrence risk varied by tumor site (highest in pNETs, 26%)	Retrospective design; incomplete pathology and recurrence data; limited follow-up for long-term survival analysis
Chen et al., 2025 [37]	The Netherlands	524 (3375 total resections analyzed) 2014–2021	68 years (60–74)	Male/Female: 265/259	Pancreas (head, body, tail)	pNET	Not reported	Not reported	Pancreatic resection (pancreatoduodenectomy or left pancreatectomy), median operative time: 258 min	Ideal outcome after pancreatic resection in 47.7% of patients, was lower than in PDAC, mainly due to a higher rate of postoperative pancreatic fistula	Retrospective registry analysis; lack of NET-specific variables (e.g., Ki-67, functionality);
Clément et al., 2025 [38]	Spain, Germany, France, Denmark, the Netherlands, Sweden, Italy, UK	251 (2014–2018)	60 years (50–67)	Male/Female: 122/129	Pancreas (body and tail)	Non-functional pNETs, mostly G1–G2	64 months for distal pancreatectomy with splenectomy) (DPS); not reached for W-SPDP or K-SPDP	Median OS not reached; no major OS difference between surgical approaches	Distal pancreatectomy with splenectomy (DPS) vs. spleen-preserving distal pancreatectomy (K-SPDP or W-SPDP), median operative time: 180 min; median follow-up: 24 months	Spleen preservation did not compromise oncologic outcomes and splenectomy did not improve lymph-node retrieval or survival after distal pancreatectomy for pNETs	Retrospective design; short median follow-up; potential selection bias in choice of surgical technique
Else et al., 2024 [39]	USA	61	41 years (19–66)	Male/Female: 32/29	Pancreas	VHL-associated pancreatic lesions, including pNETs and serous cystadenomas	Not reached, follow-up duration: median 37.8 months (range 36.1–46.1 months).	Not reported	Belzutifan (HIF-2α inhibitor) 120 mg once daily, median treatment duration: 37.3 months; median follow-up: 37.8 months	Belzutifan showed durable antitumor activity: ORR of 84% in pancreatic lesions and 91% in pNETs, with sustained responses and reduction in tumor growth rate	Single-arm design; small number of pNET patients; absence of comparator arm
Fusai et al., 2021 [40]	UK, Italy, France, Germany, the Netherlands, South Korea, Sweden, USA	541 (2007–2016)	58 years (20–85)	Male/Female: 261/280	Pancreas (head)	pNETs, G1–G3	3-year PFS: 83% (standard PD) vs. 48% (PD with vein resection); after propensity matching: 59% vs. 49%	5-year OS: 91% (standard PD) vs. 67% (PD with vein resection); after matching: 69% vs. 71%	Pancreaticoduodenectomy vs. pancreaticoduodenectomy with portal/superior mesenteric vein resection, median follow-up: 44 months	Venous resection during did not worsen perioperative risk or long-term survival, suggesting vascular involvement should not preclude surgical resection in pNETs	Retrospective design; heterogeneity between surgical centers; absence of a non-surgical comparison group
Hijoka et al., 2017 [41]	Japan	1490 (1996–2019)	62 years (54–69)	Male/Female: 978/512	Pancreas	Advanced pNETs, G1–G3	5 years: 87.3%, 10 yeas: 80.8%	5 years: 97.8% (G1), 91.8%(G2), 77.4% (G3); 10 years: 94.6% (G1), 85.0% (G2) 58.0% (G3)	Surgical resection, management based on tumor size and grade; surgery vs. surveillance, median follow-up: 4.6 years	Tumor grade/size predict metastasis and recurrence, surgery recommended for NET-G1 >20 mm, NET-G2 >10 mm, and >15 mm if grade	Retrospective design. No observational comparison group. Some biomarker/imaging data missing
Ikeda et al., 2024 [42]	Japan	74 (2017–2020)	62 years (22–89)	Male/Female: 36/38	Pancreas	pNETs, G1–G3 with liver metastases	22.9 months overall; 46.6 months (MAP-matched) vs. 15.4 months (MAP-unmatched)	47.3 months overall	Systemic therapy based on treatment-selection MAP (somatostatin analogs, molecular-targeted agents, or cytotoxic chemotherapy), study period: 2017–2020; median follow-up until June 2022	MAP-guided treatment selection improved PFS in the molecular-targeted therapy area and served as an independent favorable prognostic factor	Retrospective design; small cohort; heterogeneity of treatment choices and limited validation in the chemotherapy area
Jamison et al., 2024 [43]	USA	5 enrolled (4 evaluable) due to early termination by the drug sponsor	75 years (59–82)	Male/Female: 4/1	GEP tract (pancreas and small bowel)	Relapsed or refractory abdominal neuroendocrine tumors (NETs)	Median PFS: 381+ days (range 232+–754 days)	Median OS: 1422+ days	Entinostat (HDAC inhibitor) 5 mg orally once weekly (or 10 mg biweekly), median treatment duration: 409 days (154+–741 days	Entinostat achieved stable disease in all evaluable patients and reduced tumor growth rates by 32–83%,	Very small sample size; early trial termination by sponsor; limited statistical power
Kaltsas et al., 2022 [44]	Greece	19	Mean 55.1 years (SD 12.5)	Male/Female: 11/8	Pancreas	Metastatic pNETs, G1–G2	Median PFS: 20.4 months (95% CI 14.1–41.5)	The planned time-to-event analysis was not performed as only one death was observed during the course of the study	Everolimus 10 mg/day as monotherapy or combined with somatostatin analogues (octreotide LAR or lanreotide), mean treatment duration: 21.5 months	Everolimus showed meaningful benefit in chemotherapy-naïve metastatic pNETs patients with prolonged PFS and manageable toxicity	Small cohort size; absence of central radiological review;
Kolodziej et al., 2025 [45]	Poland	56 (interim analysis of DUONEN trial)	69 years (27–86)	Male/Female: 26/30	GEP tract (midgut 62.5%, pancreas 28.6%, others)	Well-differentiated GEP-NETs (G1–G2)	PFS not yet reported (interim safety analysis)	Will be reported upon study completion	Dosimetry-guided PRRT with [177Lu]Lu-DOTA-TATE or tandem [90Y]Y/[177Lu]Lu-DOTA-TATE, multiple cycles with individualized activity adjustment	Dosimetry-guided PRRT with individualized isotope activity modification was feasible and showed safety profile with predictable hematologic toxicity	Interim analysis with incomplete follow-up; efficacy outcomes such as PFS and OS not yet mature
Kwon et al., 2021 [46]	South Korea	918 patients who underwent curative-intent surgery across 14 tertiary	Mean 54.0 ± 12.3 years	Male/Female: 432/486	Pancreas (body/tail 58.9%, head 39.2%)	pNETs graded using WHO 2010 classification: G1 66.1%, G2 27.8%, G3 6.1%	5-year PFS: 86.5%; recurrence significantly associated with margin status, WHO grade, and lymph node metastasis	5-year OS: 91.4%; 10-year OS: 79.8%	Surgical resection, including pancreatoduodenectomy, distal pancreatectomy, enucleation, median pancreatectomy, or total pancreatectomy, not applicable—single surgical intervention	Margin positivity, higher grade (G2–G3), and lymph node metastasis were independent predictors of recurrence; tumor size >2 cm correlated with aggressive disease	Retrospective design; only resected tumors included; possible staging limitations; adjuvant treatment effects not evaluated
Maggino et al., 2025 [47]	Italy, Germany, Sweden, France, USA, and other European centers	263 patients with resected cystic pNETs	Age >65 years in 30% of patients (79)	Male/Female: 133/130	Pancreas (head 19.4%, body–tail 74.9%, diffuse 5.7%)	pNET, cystic subtype, G1 76.8%, G2 17.5%, G3 1.5%	5 years: 94.1%; 10 years: 89.5%	5-year 96.5% and 10-year OS 91.8%	Surgical resection (distal pancreatectomy 62.8%, pancreatoduodenectomy 19%, pancreas-sparing resection 15.2%, total pancreatectomy 3%), median hospital stay 8 days	Aggressive behavior in 15.6% of tumors; tumor size >2 cm, older age, and pancreatic duct dilation independently predicted aggressive disease; only 1.6% of asymptomatic tumors ≤2 cm showed aggressive behavior	Retrospective design; only surgically treated tumors analyzed; absence of centralized imaging/pathology review
Matsumoto et al., 2025 [48]	Japan	25 patients with pNETs (2020–2023)	62 years (52–71)	Male/Female: 15/10 (60% male)	Pancreas (head 44%, body 32%, tail 24%)	pNET, G1, tumor diameter ≤15 mm; all tumors nonfunctional	88% (22/25) at 6 months after treatment	6-month OS rate: 96%; one death due to cardiac infarction unrelated to tumor progression	EUS-EI with repeated injections (≤2 mL/session) using a 25-gauge needle, median procedure time 21 min, follow-up 6 months	EUS-EI achieved 76% efficacy-safety endpoint, higher than historical surgical outcomes (47.8%, *p* = 0.008); complete tumor ablation rate 88% with low severe complication rate (4%)	Non-randomized single-arm design; comparison based on historical surgical cohort; short follow-up insufficient to evaluate long-term survival
Nair et al., 2025 [49]	USA	189 patients 2010–2021	61 years (53–66 at PRRT center; 54–67 at non-PRRT center)	Male/Female: 112/79	Pancreas (head 34%, tail 45%, others reported)	pNET; G1 28%, G2 36%, G3 8%, remainder unknown	Not reported; study focused on treatment patterns rather than survival endpoints	PRRT group: 46.2%; no PRRT group: 52.9%	Multimodal therapy, including surgery (56%), somatostatin analogs (octreotide 34%, lanreotide 30%), chemotherapy (30%), PRRT (29%), targeted therapy (22%), PRRT median 4 cycles when administered	Surgery remained gold standard; PRRT was second-line therapy in stage IV disease. Availability of PRRT influenced treatment patterns between centers, with 60% PRRT use at PRRT-capable center vs. 6% at non-PRRT center	Retrospective design; treatment access differed between centers
Oziel-Taieb et al., 2021 [50]	France	48 patients with metastatic enteropancreatic NETs (2009–2018)	63 years (20–85) at first FOLFOX administration	Male/Female: 26/22	Enteropancreatic sites: pancreas 68.8%, small intestine 20.8%, unknown origin 10.4%	GEP-NETs, grades G1–G3, median Ki67 10%)	12.6 months (95% CI 10.6–16.3) based on Kaplan–Meier analysis	29.4 months (95% CI 23.9–39.4)	Modified FOLFOX-6 chemotherapy (oxaliplatin 85 mg/m^2^ + leucovorin + 5-FU bolus and infusion every 2 weeks), 6 cycles (3 months); median chemotherapy break 14.1 months before next treatment	Disease control rate 83.3% (partial response 27.1%, stable disease 56.3%). FOLFOX showed activity regardless of Ki67 index or primary tumor site and allowed short treatment duration with long chemotherapy-free interval	Retrospective design; small cohort; heterogeneous prior treatments; absence of a control group and limited statistical power for subgroup analyses
Partelli et al., 2024 [51]	Italy	31 patients with resectable non-functioning pNETs at high risk of recurrence (2020–2023)	Mean 59 ± 8 years	Male/Female: 19/12	Pancreas (head–uncinate 55%, body–tail 45%)	pNET, non-functioning, G1 (12), G2 (16); high-risk features such as tumor >4 cm, Ki-67 >10%, vascular invasion, or nodal disease	Not reported; study evaluated radiological response rather than survival endpoints	Not reported; study focused on safety and response to neoadjuvant therapy	Neoadjuvant PRRT with 177Lu-DOTATATE followed by surgical resection (pancreatoduodenectomy, distal pancreatectomy, total pancreatectomy, or atypical resection), 4 cycles of 177Lu-DOTATATE every 6–8 weeks; surgery performed ~4 months after last cycle	Partial radiologic response in 58% (18/31) and stable disease in 42% with no progression; R0 resection achieved in 24/28 patients; neoadjuvant PRRT demonstrated good safety and feasibility before surgery	No control group; small sample size; heterogeneous high-risk inclusion criteria; long-term oncologic outcomes not evaluated
Patel et al., 2025 [52]	USA	19 patients with enrolled in the SWOG S1609 DART basket trial across >1000 participating sites	62 years (18–75)	Male/Female: 10/9	Pancreas	pNETs across multiple grades (low, intermediate, and high grade)	3 months; longest responses observed with PFS up to 26–39+ months in selected responders	24 months	Dual immune checkpoint blockade: nivolumab (240 mg IV every 2 weeks) + ipilimumab (1 mg/kg IV every 6 weeks), treatment continued until progression or unacceptable toxicity	Overall response rate 11% (2/19) and clinical benefit rate 26% (partial response + stable disease >6 months). Durable responses >2 years occurred in 3 patients,	Small cohort size; heavily pretreated population; basket trial design without PNEN-specific comparator arm
Pavel et al., 2024 [53]	Germany	57	65 years (39–82)	Male/Female: 33/24	Pancreas	pNET, G1–G2 with radiologic progression prior to enrollment	15.4 months (everolimus) vs. 11.0 months (TMZ with bevacizumab)	Not reached	Everolimus 10 mg orally once daily vs. placebo, both with best supportive care, treatment continued until disease progression; treatment exposure approx. 38 weeks	Everolimus improved PFS (HR ≈ 0.35) and reduced risk of disease progression compared with TMZ with bevacizumab in patients with advanced progressive pNET	High crossover rate (≈73% of placebo patients switched to everolimus), which likely diluted the observed OS benefit
Pavel et al., 2021 [54]	Germany, France, Italy, Spain, Belgium, UK, Poland	99 patients (midgut cohort *n* = 51; pancreatic cohort *n* = 48)	67.1 ± 8.2 years (midgut) and 63.3 ± 10.6 years (pNETs)	Male/Female: 49/50 overall (midgut 29/22; pNETs 20/28)	GEP tract (midgut NETs and pNETs)	Well-differentiated NETs, G1–G2, SSTR2-positive, Ki-67 ≤20%	8.3 months (midgut NET) and 5.6 months (pNET)	OS not mature; 4 deaths occurred during follow-up	Lanreotide autogel 120 mg every 14 days after prior lanreotide 120 mg every 28 days, 48 weeks (pNETs group)	Disease control achieved in 58.8% (midgut) and 43.8% (pNETs) at 24 weeks	Single-arm design without comparator; small cohort; heterogeneous NET subtypes; OS data immature at time of analysis
Pulvirenti et al., 2021 [55]	USA, Italy, UK, Australia	632 patients in training cohort with resected pNETs, plus 328 patients in an external validation cohort	57 years (19–85) in the training cohort; 59 years (17–87) in the validation cohort	Training cohort male/female: 311/321	Pancreas (head 34%, body/tail 65%, multiple sites 1%)	Well-differentiated pNETs, G1–G2 with Ki-67 < 20%	37 months; recurrence observed in 12% of patients	not specifically reported; study focused on PFS rather than OS	Curative surgical resection, including pancreaticoduodenectomy, distal pancreatectomy, enucleation, central pancreatectomy, or total pancreatectomy, single surgical intervention; median follow-up 51 months	Developed a nomogram predicting 5-year PFS using number of positive lymph nodes, Ki-67 index, tumor size, and vascular/perineural invasion; model showed strong discrimination (c-index 0.85 training; 0.84 external validation)	Retrospective design; variability in pathological assessment across institutions; incomplete lymph node sampling in some cases
Sahara et al., 2021 [56]	USA	621 patients with distal pancreatectomy (2002–2016)	59 years (49–65) overall cohort	Male/Female: 283/338	Pancreas (body ~31%, tail ~69%)	pNET, predominantly well-differentiated (89%) or moderately differentiated (10%)	5-year: 91.1% (SPDP) vs. 84.7% (DPS)	5-year: 96.8% (SPDP) vs. 92.0% (DPS)	Surgical resection: distal pancreatectomy with spleen preservation (Warshaw technique) vs. distal pancreatectomy with splenectomy, single surgical intervention; median follow-up 35.7 months	SPDP achieved similar long-term oncologic outcomes compared with DPS while demonstrating lower blood loss, fewer complications, and shorter hospital stay	Retrospective design; potential institutional variability; lower lymph node yield with SPDP (median 3 vs. 9 nodes),
Strosberg et al., 2026 [57]	USA	24 patients enrolled in the CABINET phase III randomized trial	Cabozantinib arm: 63 (38–80); Placebo arm: 67 (47–82)	Male/Female:16/3 cabozantinib group vs. 8/3 placebo group	Pancreas, gastrointestinal tract, lung/thymus, and unknown primary	Well-differentiated G3 NETs with Ki-67 > 20%	7.9 months (cabozantinib group) vs. 3.0 months (placebo group)	OS not analyzed (data immature)	Cabozantinib 60 mg daily oral tyrosine-kinase inhibitor vs. placebo, continuous treatment until progression or toxicity	Cabozantinib improved PFS compared with placebo, produced 25% objective response rate vs. 0% with placebo	Small group sample; heterogeneous primary tumor sites; pretreated population
Xu et al., 2025 [58]	China	163 patients with hepatic metastases (1996–2019)	<60 years (73%) vs. ≥60 years (27%)	Male/Female: 83/80	Pancreas with synchronous liver metastases	pNETs, G1–G3	Not reported	69 months; by treatment: 122 months (primary + hepatic resection), 72 months (primary resection only), 32 months (no surgery)	Surgical strategies: pancreatic resection (PR), combined pancreatic and hepatic resection (PHR), or non-surgical management, approximately 48 months follow-up	Combined resection of primary tumor and liver metastases significantly improved survival particularly in tumors located in the pancreatic body/tail	Retrospective design, heterogeneous treatment, potential selection bias, long study period with evolving diagnostic and therapeutic techniques
Zhang et al., 2021 [59]	USA	854 with curative-intent resection	58 years (47–66)	Male/Female:412/442	Pancreas	pNETs, G1–G3	5-year: 82.6% (node-negative), 63.2% (1–3 nodes), 43.5% (≥4 nodes)	5-year: 91.3%(N0), 87.5% (N1), 67.0% (N2) in institutional cohort; SEER validation 84.2% vs. 75.0% vs. 62.6%	Curative surgical resection (distal pancreatectomy, pancreaticoduodenectomy, or enucleation) with lymphadenectomy, single surgical intervention; median follow-up 35 months	Metastatic lymph nodes predicted recurrence and survival; ≥4 positive nodes associated with significantly worse prognosis; evaluation of ≥8 lymph nodes improved staging accuracy	Heterogeneity in lymphadenectomy extent; lack of standardized lymph node station assessment

**Table 4:** Pearson’s correlation analysis between study sample size and PFS.

		Participants	PFS_Months	OS_Months
Participants	Pearson’s Correlation	1	0.592 *	−0.054
	Sig. (2-tailed)		0.026	0.909
	*N*	27	14	7
PFS_months	Pearson’s Correlation	0.592 *	1	0.245
	Sig. (2-tailed)	0.026		0.640
	*N*	14	14	6
OS_months	Pearson’s Correlation	−0.054	0.245	1
	Sig. (2-tailed)	0.909	0.640	
	*N*	7	6	7

* Correlation is significant at the 0.05 level (2-tailed).

**Table 5:** Spearman’s correlation analysis between study sample size, PFS, and OS.

			Participants	PFS_Months	OS_Months
Spearman’s rho	Participants	Correlation Coefficient	1.000	0.744 **	0.214
		Sig. (2-tailed)	.	0.002	0.645
		*N*	27	14	7
	PFS_months	Correlation Coefficient	0.744 **	1.000	0.600
		Sig. (2-tailed)	0.002	.	0.208
		*N*	14	14	6
	OS_months	Correlation Coefficient	0.214	0.600	1.000
		Sig. (2-tailed)	0.645	0.208	.
		*N*	7	6	7

** Correlation is significant at the 0.01 level (2-tailed).

## Supplementary Materials

The following supporting information can be downloaded at: https://www.mdpi.com/article/10.3390/cancers18111705/s1.
