# Supplementary material for: Survival Outcomes in Pancreatic Neuroendocrine Tumors: A Systematic Review and Meta-Analysis of Progression-Related Endpoints
Source: Cancers (Basel). 2026 May 23;18(11):1705. doi: 10.3390/cancers18111705 (PMC13256032; doi:10.3390/cancers18111705)
Supplement: Supplementary file 1 [file cancers-18-01705-s001.zip › cancers-4275910-supplementary material-xml.pdf]

cancers

Supplementary Materials: Survival Outcomes in Pancreatic Neuroendocrine Tumors: A Systematic Review and Meta-Analysis of Progression-Related Endpoints

Lavinia Simona Neculai-Candea , Andreea-Daniela Caloian, Sorin Deacu, Miruna Cristian, Laura Mazilu, Andreea-Corina Ilie-Petrov, Radu Adrian Nitu, Carmen Aida Ciufu and Nicolae Ciufu

Table S1. Newcastle–Ottawa Scale quality assessment of studies included in the meta-analysis.

Study	Study Design	Selection (0–4)	Comparability (0–2)	Outcome (0–3)	Total NOS Score	Quality
Addeo et al. 2024	Retrospective multicenter cohort	4	2	2	8	High
Chan et al. 2025	Randomized placebo-controlled trial	4	2	3	9	High
Fusai et al.	Retrospective cohort	3	1	2	6	Moderate
Ikeda et al.	Retrospective comparative study	3	2	2	7	High
Kaltsas et al.	Retrospective cohort	3	1	2	6	Moderate
Kwon et al.	Retrospective cohort	3	1	2	6	Moderate
Matsumoto et al. 2025	Prospective multicenter study	4	1	3	8	High
Oziel-Taieb et al.	Retrospective observational study	3	1	2	6	Moderate
Partelli et al. 2024	Prospective phase II multicenter trial	4	1	3	8	High
Pavel et al. 2021	Randomized clinical trial	4	2	3	9	High
Pulvirenti et al.	Retrospective cohort	3	1	2	6	Moderate
Strosberg et al. 2026	Phase III randomized trial	4	2	3	9	High
Zhang et al. 2021	Multi-institutional retrospective analysis	4	1	2	7	High

NOS: Newcastle–Ottawa Scale. Studies scoring 7–9 points were considered high quality, 5–6 moderate quality, and <5 low quality.
